# Propofol-based palliative sedation in terminally ill children with solid tumors: A case series

**DOI:** 10.1097/MD.0000000000015615

**Published:** 2019-05-24

**Authors:** Evelina Miele, Angela Mastronuzzi, M. Giuseppina Cefalo, Francesca Del Bufalo, M. Debora De Pasquale, Annalisa Serra, Gian Paolo Spinelli, Luigi De Sio

**Affiliations:** a Department of Hematology/Oncology and Stem Cell Transplantation, Bambino Gesù Children's Hospital, Istituto di Ricovero e Cura a Carattere Scientifico (IRCCS); b Unità Operativa Complessa Oncology, University of Rome “Sapienza”, Azienda Sanitaria Locale Latina District 1, Aprilia (LT), Rome, Italy.

**Keywords:** childhood, end of life, pain, pediatric palliative sedation, propofol, solid tumors

## Abstract

**Rationale:**

The palliative sedation therapy is defined as the intentional reduction of the alert state, using pharmacological tools. Propofol is a short-acting general anesthetic agent, widely used for induction and maintenance of general anesthesia and rarely employed in palliative care.

**Patient concerns and Diagnoses:**

This case series describes 5 pediatric oncology inpatients affected by relapsed/refractory solid tumors received palliative sedation using propofol alone or in combination with opioids and benzodiazepines.

**Interventions and Outcomes:**

Five terminally ill children affected by solid tumors received propofol-based palliative sedation. All patients were previously treated with opioids and some of them reduced the consumption of these drugs after propofol starting. In all cases the progressive increase of the level of sedation until the death has been the only effective measure of control of refractory symptoms related todisease progression and psychological suffering.

**Lessons:**

We evaluated the quality of propofol-based palliative sedation in a series of pediatric oncology patients with solid tumors at the end of their life. We concluded that propofol represents an effective and tolerable adjuvant drug for the management of intractable suffering and a practicable strategy for palliative sedation in pediatric oncology patients at the end of their life.

## 1 Introduction

About 80% of pediatric patients with cancer in terminal phase experience pain refractory to the traditional analgesia, together with psychological and existential suffering.^[1]^ The term of “refractory symptoms” refers to a more general suffering subjective feeling of the terminally ill child. Classical pharmacological analgesia, based on opioids and benzodiazepines, allows keeping under control the pain in <30% of cases.

The limited knowledge and experience of the clinicians about painkillers dosages and medications in pediatric age, the fear of side effects and possible narcotic addiction and the difficulty in assessing and grading pain, lead to inadequate control of symptoms in pediatric cancer patients^[2]^ at the end of their life.

The palliative sedation therapy is defined as the intentional reduction of the alert state, using pharmacological tools, up to the lost of consciousness, with the aim of decrease or eliminate a symptom perception or a psychological mood, otherwise not tolerable for the patient. The palliative sedation therapy can be addressed to pediatric cancer patients at the end of their life experiencing intractable pain, restlessness, agitation, psychological suffering, refractory to standard management. The primary aim should be to induce light to deep sleep, reaching a state of appeasing sedation. Midazolam, neuroleptics, and barbiturates are the drugs most commonly used.^[3]^ In adult cancer patients, in case of failure of the standard measures of symptoms control, the addition of the anesthetic propofol may represent a feasible strategy to ameliorate pain control.^[4]^ Propofol is a short-acting general anesthetic agent, widely used for induction and maintenance of general anesthesia and rarely employed in palliative care.^[5]^ It presents a short onset of action and, if given in continuous infusion, its plasma level can be titrated in order to modulate the deepness of sedation and the low context-sensitive half-life. Propofol non-sedative effects include antiemetic, bronchodilatation, and itching control. It also exhibits potentially severe side effects, such as hypotension, dysrhythmias, respiratory depression, and multi-system organ failure. In pediatric terminal cancer patients, its use has been pointed out only in anecdotal reports.

We present the experience of the Pediatric Hemato-Oncology Department at the Bambino Gesù Children's Hospital in using propofol to obtain palliative sedation in a case series of 5 terminally ill children affected by solid tumors.

## 2 Patients and methods

We retrospectively recorded the data of all children affected by solid tumors who received palliative sedation with propofol from 2006 to 2012 at the Pediatric Hemato-Oncology Department at the Bambino Gesù Children's Hospital.

Aim of propofol addition to conventional drugs was to achieve the control of refractory clinical symptoms and unbearable distress, experienced at the end of life, by reaching an adequate level of sedation. Cases of patients treated with propofol during invasive procedures or for other indications, like refractory nausea and vomiting, were excluded. Our institutional review board did not require its approval for this type of intervention and parent's patient informed consent for treatments and publication of the case details was obtained. Moreover all the procedures were performed in accordance with Declaration of Helsinski 2013.

Data of the pediatric cancer patients ongoing palliative sedation therapy at our Institution were detailed. Patients’ data collection included: age, sex, diagnosis, oncologic treatment (surgery, chemotherapy, radiotherapy), and disease status (Table 1). A trained team composed by physicians and nurses closely monitored all patients. Pain intensity was evaluated using numeric rating scales according to the age of patients, as Face-Legs-Activity-Cry-Consolability scale (FLACC) scale for age <3 years and visual analogue scale (VAS) for age >3 years; when a satisfactory grade of sedation was obtained, the changes in the heart rate, blood pressure, pulse oxymetry, and respiratory rate were monitored as indirect parameters of patient's discomfort.

**Table 1:**
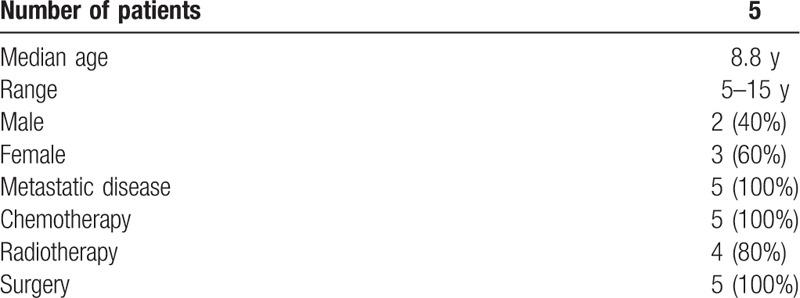
Patient data.

Palliative sedation therapy details were considered: indication to start propofol treatment, dose of propofol (starting dose, dose-ranging, and final dose), duration of treatment, and reason to stop it. Propofol (10 mg/mL; Diprivan, Astra Zeneca, Sweden) was infused through computerized infusion pomp. The titration of propofol was performed in steps of 0.5 mg/kg/h. All drugs given 24 hours before starting the infusion with propofol were recorded. All patients had a central venous catheter for therapy. Sedation status, defined as awake versus asleep, was evaluated from 24 hours before propofol starting, up to the patient's death. Adverse events related to propofol use, as agitation, hallucinations, twitching, seizures, and the propofol-related infusion syndrome, were also monitored. Data received the Internal Review Board endorsement.

## 3 Results

From 2006 to 2012, in our Department, 5 pediatric terminal cancer patients underwent palliative sedation with propofol.

The indications for using propofol were disease relapsed and/or progressed and refractory to oncologic treatment, pain, agitation, and psychological suffering refractory to conventional treatment.

Patient demographic and clinical data are summarized in Table 1. Patients were 2 men and 3 women. The median age was 8.8 years (range, 5–15). All patients were affected by metastatic solid tumors: 2 neuroblastoma with bone marrow metastases, 1 nephroblastoma with pulmonary and peritoneal metastases, 1 Ewing sarcoma with pulmonary metastases, and 1 NUT-midline carcinoma of the pancreas with hepatic dissemination. All patients had previous received multidisciplinary anticancer treatments: 5/5 patients underwent surgical excision/biopsy of primary tumor and at least 2 lines of chemotherapy; radiotherapy was performed in 4/5 patients. At the moment of admission at our Unit, all the patients presented disease progression and suffered for uncontrolled pain. All patients had a central venous access for treatment.

The starting dose of propofol ranged from 0.3 to 2 mg/kg/h, according to the grade of sedation. In case of unsatisfactory sedation level, dose of propofol was increased based on patient's clinical needs. During the overall treatment period, the dose range was between 0.3 and 16 mg/kg/h (mean 4.5 mg/kg/h), with an adequate level of sedation at dose between 3 and 16 mg/kg/h in the most of patient. The mean length of treatment duration was 4.4 days (Table 2).

**Table 2:**
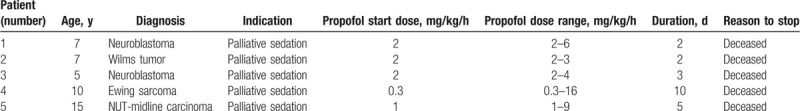
Details of propofol treatment.

Before starting propofol all patients were previously treated with strong opioids combinations and received continuous intravenous infusion of midazolam, without symptom relief (Table 3). Two out of 5 patients required continuous infusion of morphine until dose of 0.06 mg/kg/h; patient-controlled-analgesia (PCA) system was adopted in 2 patients. One child experienced continuous intrathecal infusion of ropivacaine. Furthermore, one patient received 1.5 mg/kg/d continuous infusion of ketamine associated to morphine. In one case thiopental infusion was started and associated with propofol to strengthen the effectiveness of sedation.

**Table 3:**
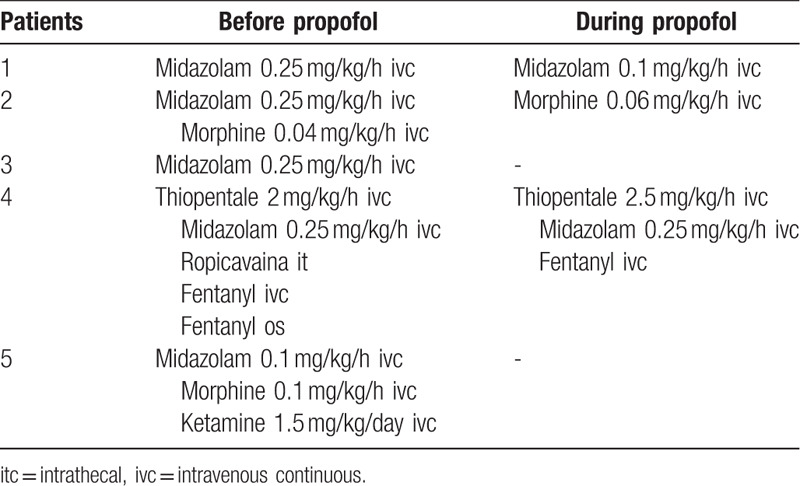
Details of other drugs administered before and during propofol treatment.

The doses of midazolam varied from 0.1 to 0.25 mg/kg/h (Table 3). Two patients needed continuous midazolam infusion concurrently with propofol, while 1 patient received only occasional doses of midazolam during propofol infusion.

All patients required continuous infusion of propofol and reached a desired level of sedation until death, with a reduction of opioids consumption and other supportive medications (benzodiazepine-midazolam).

A multidisciplinary team composed by oncologists, pallitivists, anesthesiologysts, psicologysts, and nurses closely monitored all patients and support their family members and caregivers. Pain intensity was evaluated using the visual analogue scale (VAS) for age >3 years; also monitoring patient's cardiac and pulmonary parameters.

None experienced adverse effects related to propofol use or developed a propofol-related infusion syndrome. At the end of their life patients were receiving propofol at a dose ranged from 3 to 16 mg/kg/h associated with midazolam in 2/5 patients, with morphine in 1/5 patients and with fentanyl in 1/5 patients.

All 5 patients died at the Unit at a median time of 4.4 days (range, 2–10) from starting propofol.

## 4 Discussion

The first aim of palliative sedation for patients with incurable disease is suffering relief.^[6]^ Sedative drugs should be adapted and monitored in relation to the deepness, continuity, and duration of sedation required to obtain this effect. The most common refractory symptoms requiring palliative sedation are delirium, dyspnea, psychological distress and intractable pain.^[7]^ The appearance of these symptoms in cancer patients generally precedes the death at short time.^[8]^ In pediatric setting, increasing psychological distress has been reported to be a strong indication to start palliative sedation^[8,9]^ and Wolfe et al^[10]^ highlighted that it must be considered a substantial aspect of the global suffering of a pediatric cancer patient, mostly in the last month of life. In our experience, palliative sedation was started as it unequivocally represented the only reasonable option to control refractory symptoms.

Both components of patient's pain and suffering, physical and psychological, were strongly considered in the hard proposal of the palliative sedation. The decision was discussed and shared with the families in all the cases. The choice to start propofol was based on disease status and severity of patients’ symptoms.

Propofol is a short-acting intravenous sedative-hypnotic agent^[11]^ formulated in a white, oil-in-water emulsion that usually produces hypnosis within 1 minute from starting intravenous administration. Propofol is extremely useful in general anesthesia, due to its short half-life and small accumulation in the body. Moreover propofol is commonly used for procedural sedation with numerous studies that found the importance of loading dose of 2 mg/kg followed by a continuous infusion demonstrate a better outcome in terms of postoperative behavioral disturbance.^[12]^ The Propofol mechanisms of action^[13]^ include prolongation of synaptic inhibition by positive modulation of gamma-aminobutyric acid type A (GABA_A_) receptor function; propofol interacts with an allosteric site on the GABA_A_ receptor, potentiates the currents elicited by low concentrations of GABA, increases agonist efficacy, and modulates receptor desensitization. Only one or all of these effects on inhibitory GABA-mediated (GABAergic) and excitatory glutamatergic synaptic transmission may contribute to the ability of propofol to produce hypnosis, amnesia, immobility and/or unconsciousness. In pediatric oncology, its use has been standardized in procedural pain control and to obtain sedation during short-term surgical procedures.^[14]^ In particular, Ince et al^[15]^ suggested that during short hemato-oncologic procedures in children, such as lumbar puncture or bone marrow aspiration, the propofol–remifentanil combination is suitable for deep sedation and early recovery.

Considering a large intra-individual variability of the propofol dosage for sedation induction in children with cancer, the drug levels are easily titratable, until a sufficient sedation is reached.^[16]^ In addition, due to its anesthetic properties, propofol acts as an antiemetic, representing an ideal drug for cancer pain associated with nausea and vomiting.^[17]^

When used in combination with opioids, propofol adequately controls discomfort induced by these drugs, such as constipation, nausea, and vomiting. However, it has been reported that propofol may increase, in combination with opioids, the rate of adverse events, such as bradycardia, hypotension, and tremors or twitching.^[18]^ Cornfield et al^[19]^ reported the safe use of continuous infusion of propofol for sedation in critically ill pediatric patients: the dose should not exceed 4 mg/kg/h and the duration of infusion should not exceed 48 hours. In our case series, the mean dose administered was 4.5 mg/kg/h and the mean duration of infusion exceeded 100 hours.

Moreover, in critically ill children who received continuous infusion of propofol for sedation, the development of a propofol-related infusion syndrome (PRIS) has been described.^[20]^ This life-threatening syndrome^[21]^ is characterized by progressive metabolic acidosis, hyperlipemia, hepathomegaly, rhabdomyolisis, and hemodynamic instability culminating in cardiovascular collapse.

Propofol-related infusion syndrome (PRIS) is a rare complication that occurs after prolonged infusion of propofol. In a long series of studies children death are reported after receiving long term and/or high dose of propofol infusion due to myocardical failure, rhabdomyolysis, and metabolic acidosis.^[1,22,23]^

In our patients, we strictly monitored clinical features, and laboratory findings and none of them experienced PRIS. Anghelescu et al^[24]^ reported that during propofol-based palliative sedation, in 3 children with terminal oncologic disease, the consumption of opioids and other supportive medications decreased. Our case series is in line with the literature data, since opioids consumption was decreased in 4/5 patients.

We considered the use of propofol with the aim of sedation, in patients poorly responding to benzodiazepines. The analgesic effect of benzodiazepines is well known while their sedation properties are less known and studied. The concomitant use of propofol seems to act in synergy with the sedation induced by benzodiazepines.^[25]^ In literature there are only few single reports and a unique largest series, composed by 9 pediatric patients with diagnosis of hematologic or solid tumors, focusing on the pediatric palliative sedation.^[26]^ Glover et al^[27]^ reported the propofol addition to hydromorphone infusion for 10 days in a 3-year-old female patient with disseminated rhabdomyosarcoma. Tobias^[28]^ reported concurrent hydromorphone and propofol infusion for 16 hours in a 14-year-old female patient affected with advanced T-cell lymphoma.

At the best of our knowledge, this is one of the first series of pediatric patients affected by solid tumors at the end of life in which we experienced the effectiveness of palliative sedation therapy using propofol. The pediatric palliation should be evaluated in all components as physical, psychosocial, and spiritual. Nonetheless, sedation in terminally ill pediatric patients should not be associated with hastening of death.^[29]^ As in the Hooke's et al^[27]^ series, we conclude that propofol represents an effective and tolerable adjuvant drug for management of intractable pain and for palliative sedation in pediatric oncology patients. However, the effective pain control and suffering relief in terminal pediatric cancer patients remains a hard challenge for clinicians. Considering the age of the patients, in our series we have constantly monitored the VAS scale demonstrating that and none of the children during the treatments showed a scale value higher than medium-low. Most large pediatric oncology series are needed to systematize the role of propofol in pediatric palliative sedation.

## Author contributions

**Supervision:** Luigi De Sio.

**Visualization:** Angela Mastronuzzi, M. Giuseppina Cefalo, Francesca Del Bufalo, M. Debora De Pasquale, Annalisa Serra.

**Writing – original draft:** Evelina Miele, Luigi De Sio.

**Writing – review & editing:** Evelina Miele, Gian Paolo Spinelli, Luigi De Sio.

## Correction

When originally published, Drs. Angela Mastronuzzi, Annalisa Serra and Luigi De Sio's name appeared incorrectly with first and surnames reversed. This has since been corrected.
